# *Alpinia zerumbet* leaf extract reverses hypertension and improves adverse remodeling in the left ventricle and aorta in spontaneously hypertensive rats

**DOI:** 10.1590/1414-431X2024e14210

**Published:** 2025-02-03

**Authors:** M.P. Menezes, G.P. Santos, D.V.Q. Nunes, D.L.B. Silva, C.P. Victório, C. Fernandes-Santos, G.F. de Bem, C.A. Costa, A.C. Resende, D.T. Ognibene

**Affiliations:** 1 Departamento de Farmacologia, Instituto de Biologia, Universidade do Estado do Rio de Janeiro, Rio de Janeiro, RJ, Brasil; 2 Laboratório de Pesquisa em Biotecnologia Ambiental, Programa de Pós-Graduação em Ciência e Tecnologia Ambiental, Universidade do Estado do Rio de Janeiro, Rio de Janeiro, RJ, Brasil; 3 Departamento de Ciências Básicas, Universidade Federal Fluminense, Nova Friburgo, RJ, Brasil

**Keywords:** Alpinia zerumbet, Spontaneously hypertensive rats, Hypertension, Cardiovascular remodeling, Oxidative stress

## Abstract

*Alpinia zerumbet*, a plant native to East Asia, is widely found on the Brazilian coast, where it is used in folk medicine as an antihypertensive, diuretic, and anxiolytic. This study investigated the effects of the hydroalcoholic extract obtained from *Alpinia zerumbet* leaves (AZE) on cardiovascular changes and oxidative status in spontaneously hypertensive rats (SHR). SHR and Wistar-Kyoto male rats, 90 days old, treated or not with AZE (50 mg/kg/day in drinking water) for six weeks, were used in this study. Blood pressure (BP) was assessed weekly by tail plethysmography. At the end of treatment, the animals were anesthetized with thiopental (70 mg/kg, *ip*), blood was collected through abdominal aorta puncture, the thoracic aorta and left ventricle were isolated for morphometric analysis and immunostaining of NOX-4, SOD-2, 8-isoprostane, and angiotensin II AT_1_ receptors (AT1R), and the mesenteric arterial bed (MAB) was isolated for the assessment of vascular function. Oxidative damage in lipids and proteins and the enzymatic antioxidant activity were evaluated in plasma samples by spectrophotometry. AZE normalized BP in SHR. Although the treatment did not improve the MAB vascular dysfunction, it reversed the cardiovascular remodeling in the aorta and left ventricle. In addition, AZE improved antioxidant activity in plasma and SOD-2 immunostaining in the thoracic aorta and left ventricle, decreased protein carbonylation in plasma, and reduced 8-isoprostane, NOX-4, and AT1R immunostaining in the cardiovascular system. The results suggested that AZE reversed hypertension and cardiovascular remodeling in SHR, which was associated with lower oxidative stress and AT1R.

## Introduction

Hypertension remains the most prevalent risk factor for cardiovascular diseases (CVD), including heart failure, coronary artery disease, stroke, myocardial infarction, atrial fibrillation, and peripheral artery disease. It is also the leading cause of CVD morbidity and mortality (1). Although the pharmacological treatment of hypertension is well established, most patients require two or more anti-hypertensive medications to achieve blood pressure goals, which also increases the risk of side effects and medication costs, reducing adherence to conventional therapies (2). In this context, over the last decades, research has focused on plants as possible sources of bioactive products with therapeutic value, minimal side effects, and low cost.

*Alpinia zerumbet* (K. Schum) is an aromatic plant originating from East Asia, also abundant on the Brazilian coast, where it is known as “Colônia” (3). Infusions or decoctions of *A. zerumbet* leaves are commonly used in folk medicine for their anti-hypertensive and diuretic properties (4). Several cardiovascular actions of essential oil and extracts from the *A. zerumbet* leaves have been reported in animal models, such as endothelium-dependent vasodilation (3,5), anti-hypertensive effect in DOCA-salt hypertensive rats (5,6), and cardioprotection in isoproterenol-induced myocardial infarction (7). However, the effects of *A*. *zerumbet* on cardiovascular changes in spontaneously hypertensive rats (SHR), an experimental model of essential hypertension, are not yet known.

Oxidative stress is well-characterized in hypertension and occurs either by increased reactive oxygen species (ROS) production or decreased antioxidant activity (8). Renin-angiotensin system (RAS) activation is essential in hypertension-related oxidative stress and cardiovascular remodeling. Angiotensin II (Ang II), the main effector of RAS, interacts with the Ang II type 1 receptor (AT1R) and activates the enzyme nicotinamide adenine dinucleotide phosphate (NADPH) oxidase increasing ROS generation, which is related to oxidative damage in the cardiovascular system (9). Oxidative damage induces tissue fibrosis and vascular smooth muscle cell (VSMC) growth, proliferation, and migration (10). Moreover, ROS negatively affect myocardial calcium concentrations, cause arrhythmia, and contribute to cardiac remodeling by inducing hypertrophic signaling, apoptosis, and necrosis (11). In addition, excess superoxide anion production scavenges nitric oxide (NO), a critical vasodilator produced by endothelial cells, contributing to endothelial dysfunction (12).

In this study, we investigated whether chronic treatment with the hydroalcoholic extract from *A*. *zerumbet* leaves (AZE) reverses hypertension, endothelial dysfunction, and cardiovascular remodeling in SHR, a commonly employed model of essential hypertension. We also investigated the modulation of RAS components and oxidative stress as possible mechanisms involved in the beneficial cardiovascular effects of AZE.

## Material and Methods

### *Alpinia zerumbet* leaves extract

Leaves of *A*. *zerumbet* were collected in Rio de Janeiro, RJ, Brazil (22°54'24.65''S.43°10'22.43''W). Plants were authenticated and a voucher specimen was deposited in the Herbarium Prof. Jorge Pedro Pereira Carauta of the Universidade Federal do Estado do Rio de Janeiro (UNIRIO), under the number HUNI5015.

Leaves underwent a natural drying process for one week through exposure to air at room temperature (20-22°C). AZE was obtained by weighing 50 g of dried leaves and boiling them in 400 mL of distilled water for 10 min. After cooling, the decoction was extracted by adding 400 mL of ethanol. The leaf suspension was stored in amber bottles at 4°C for 10 days, with 1 h of daily stirring (Kline New Technique model NT 150, Brazil). The obtained extract was filtered through Whatman #1 filter paper, and ethanol was evaporated (Fisatom Equipamentos Científicos Ltda., Brazil) under low pressure at 65°C, for further freeze-drying (LIOTOP model 202, Brazil) at -40°C under 200 mmHg vacuum and maintained at 4-8°C until use.

AZE was analyzed by UHPLC/ESI-QTOF-MS and the GNPS spectral libraries identified the following flavonoids: (epi)catechin, procyanidin B2, quercetin-3-O-glucuronide (Q3OG), kaempferol-3-O-glucoside-3”-rhamnoside (K3OG3R), kaempferol-3-O-glucuronide (K3OG), isorhamnetin-3-O-neohesperidoside (I3ON), alpinetin, and pinocembrin (13).

### Animals

This study was reviewed and approved by the Ethics Committee of Animal Experiments of the Rio de Janeiro State University (CEUA/52/2016). The investigation followed the principles of the “Guide for the Use and Care of Laboratory Animals” from the National Institute of Health (NIH) (14), as well as the principles of the “Brazilian Guide for the Production, Maintenance, or Use of Animals in Teaching or Scientific Research Activities” from the National Council for the Control of Animal Experimentation (CONCEA). The animals were housed under standardized conditions with an appropriate temperature (21±2°C), a light-controlled cycle (dark-light cycles of 12 h with light cycle from 6:00 a.m.), and had free access to water and rodent chow (Nuvilab^®^, Brazil). Three-month-old male Wistar Kyoto (WKY) and SHR were assigned to four groups: WKY, WKY+AZE, SHR, and SHR+AZE (n=10 per group). AZE (50 mg/kg/day) (5) was administered in the drinking water for six weeks. Three to four rats of the same group were kept per cage. Before treatment, water consumption per day was estimated in each cage. During treatment, water and AZE offerings were adjusted every two days according to the water consumption and the animals' weight in each cage.

Systolic, diastolic, and mean blood pressures were measured in conscious rats once a week by tail-cuff plethysmography (CODA-HT2, Kent Scientific^®^, USA).

After six weeks of treatment, rats were housed in metabolic cages to collect 24-h urine for volume quantification. Then, animals were anesthetized (thiopental sodium 70 mg/kg, intraperitoneally), and blood was collected by aorta puncture, with subsequent isolation of the mesenteric arterial bed (MAB), thoracic aorta, and heart.

### Vascular perfusion studies

MABs were isolated by the method described by McGregor (15). Briefly, after blood collection, MAB was rapidly removed, cannulated, and perfused at a flow rate of 4 mL/min with a physiological salt solution (PSS) using a peristaltic pump (Model MINIPLUS 3, Gilson^®^, USA). The PSS (in mmol/L: 118 NaCl, 4.7 KCl, 2.5 CaCl_2_, 1.2 MgSO_4_, 1.2 KH_2_PO_4_, 25 NaHCO_3_, 0.026 EDTA, and 6.0 glucose) was bubbled with 95% O_2_/5% CO_2_ at 37°C. Perfusion pressure (PP) was measured using a pressure transducer (PowerLab 4/30) and continuously registered on a computer through the LabChart 7 reader program (ADInstruments, New Zealand). The drugs were either dissolved in PSS and perfused at the desired concentration or administered as bolus injections directly into the perfusion stream, close to the arterial cannula. The preparations were left to equilibrate for 30 min, and 120 µmol KCl was administered every 10 min until consistent responses were obtained. Then, basal perfusion pressure was elevated (80-100 mm Hg) by adding norepinephrine (NE; 30 µM) to the perfusion solution. After the vasopressor response of NE reached a constant plateau, acetylcholine (ACh; 1-300 pmol) was injected, and the vasodilator effect was measured as the percentage decrease in the pressor effect of NE. After washing the preparations with PSS for 20 min, the vasoconstrictor response to NE (1-1,000 nmol) was assessed by bolus injections and reported as % increase in basal PP (mmHg).

### Oxidative damage, antioxidant activity, nitrite, and Ang II levels

Oxidative damage and the activity of antioxidant enzymes were determined in plasma samples. The formation of lipid peroxidation products (malondialdehyde, MDA) and protein carbonylation were assayed to determine the oxidative damage, as previously described (16,17). Superoxide dismutase activity was assayed by measuring the inhibition of adrenaline auto-oxidation as absorbance at 480 nm (18). Catalase activity was measured as the rate of decrease in hydrogen peroxide at 240 nm (19). Glutathione peroxidase activity was measured by monitoring the oxidation of NADPH at 340 nm in the presence of hydrogen peroxide (20). Total protein content in each sample was determined using the Bradford method (21).

NO content was estimated in plasma samples by the formation of nitrite (NO_2_) using the Griess method (22). Plasma levels of Ang II were determined by an ELISA kit (Elabscience^®^, USA).

### Aorta remodeling

The thoracic aorta was fixed in 4% buffered formalin pH 7.2 for 48 h, embedded in Paraplast plus (Sigma-Aldrich Co., USA), and sectioned (3-µm-thick sections; Leica Rotary Microtome, Leica Biosystems, Germany). Six non-consecutive aorta transverse sections per animal were mounted on glass slides and stained with hematoxylin-eosin for morphometry or Picrosirius red to analyze collagen deposition. Digital images were acquired (JPEG format, 36-bit color, 1,280×1,024 pixels) with an Olympus LC Evolution camera (Japan) and an Olympus BX51 light microscope (Japan) and analyzed with the software ImageJ (version 1.54e, NIH, USA).

Tunica media thickness was defined by the region delimited by the internal and external elastic lamina, and four measurements per image were obtained at 0°, 90°, 180°, and 270° (magnification: ×20) using the length tool. For lumen area quantification, one measure per image was obtained (×4 magnification) using the polygon tool (23).

To estimate the collagen area (%) of fibrosis, ten random images were obtained per animal (×20 magnification) and analyzed using the color deconvolution tool. The mean value was subsequently calculated (23).

### Left ventricle remodeling

The heart was removed, and the left ventricle (LV) was carefully dissected to evaluate cardiac and LV hypertrophy. LV samples were immersed in 4% buffered formalin pH 7.2 for 48 h. To determine the LV hypertrophy, LV samples were cut transversally at their half-height based on the base-apex axis, and a slice underwent routine histological processing, embedded in Paraplast plus (Sigma-Aldrich Co.), cut 5-µm thick, and stained with hematoxylin-eosin. Four to six animals per group and three non-consecutive sections per animal were used. Images of the LV chamber were acquired with a stereomicroscope (×0.5 magnification; SteREO Discovery.V8, CL 6000 LED, Zeiss, Germany) and analyzed with the software ImageJ (version 1.54e, NIH). The LV free wall thickness and the interventricular septum thickness at the level of the papillary muscles were assessed using the length tool. The lumen area of the LV chamber was assessed using the polygon tool (24).

To estimate the collagen area (%) of fibrosis, samples of the apex of the LV were cut, stained with Picrosirius red, and a quantitative examination was carried out on a light-microscope (×20 magnification; LC Evolution camera and an Olympus BX51). For each heart, six images were randomly selected and analyzed using the color deconvolution tool. The mean value was subsequently calculated (25).

### Immunohistochemistry

Sections of the thoracic aorta (3 µm) and LV (5 µm) were deparaffinized, rehydrated, and incubated (15 min) with 3% H_2_O_2_ to block endogenous peroxidase. Nonspecific protein binding was blocked by incubation with 2.5% bovine serum albumin diluted in phosphate-buffered saline with bovine serum albumin (PBS/BSA). Antigen retrieval was performed with trypsin (3%) diluted in distilled water for 10 min at 37°C or citrate buffer for 20 min at 60°C. The sections were incubated with the primary antibody at 1:100 dilution with 1% PBS/BSA overnight (4°C in a humid atmosphere). This procedure identified the following proteins: AT1R (1:100 dilution, NBP1-77078, Novus Biologicals, USA), 8-isoprostane (1:100 dilution, IS20, Oxford Biomedical Research, USA), NOX-4 (1:50 dilution, sc-30141, Santa Cruz Biotechnology Inc., USA), and superoxide dismutase 2 (1:100 dilution, sc-30080, Santa Cruz Biotechnology Inc.). The signal was amplified with a biotin-streptavidin complex system (Vectastain Universal Quick kit; Vector Laboratories, USA), and the positive immunoreaction was identified after incubation with 3,3' diaminobenzidine tetrachloride (DAB; Dako Cytomation, Denmark). Sections were counterstained with hematoxylin for cell nuclei identification and the slides were then mounted. For each rat, six images per tissue were randomly selected and analyzed with the software Image J using the color deconvolution tool (version 1.54e, NIH).

### Statistical analysis

Data are reported as means±SE. The normality of the data was verified using the Shapiro-Wilk test. Two-way ANOVA or three-way ANOVA followed by Sidak *post hoc* test was used to compare differences among experimental groups. Data were analyzed using GraphPad Prism 6.0.1 (GraphPad Software Inc., USA). P-values less than or equal to 0.05 were accepted as statistically significant.

## Results

### Effects of AZE on blood pressure measurements and 24-h urine volume

**Figure 1:**
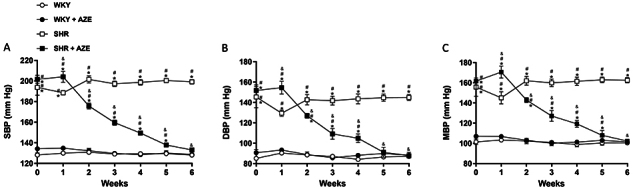
Arterial blood pressure measurements. Systolic (**A**), diastolic (**B**), and mean (**C**) blood pressure. Data are reported as means±SE, n=9-10 per group. *P<0.05 *vs* WKY group; ^#^P<0.05 *vs* WKY+AZE group; ^&^P<0.05 *vs* SHR group (three-way ANOVA followed by Sidak *post hoc* test). AZE: *Alpinia zerumbet* leaf extract; WKY: Wistar Kyoto rats; SHR: spontaneously hypertensive rats; SBP: systolic blood pressure; DBP: diastolic blood pressure; MBP: mean blood pressure.

As shown in Figure 1, systolic, diastolic, and mean blood pressures were significantly higher (P<0.0001) in the SHR groups than in the controls at the beginning of the experimental protocol. AZE treatment reduced (P<0.0001) the blood pressure in the SHR+AZE group from the second week of treatment, reaching the levels of the controls by the sixth week.

On the other hand, 24-h urine volume did not differ among experimental groups (mL/24 h, WKY: 9.9±0.99; WKY+AZE: 8.5±0.53; SHR: 11.2±0.77; SHR+AZE: 9.3±0.42).

### Effects of AZE on vascular reactivity

**Figure 2:**
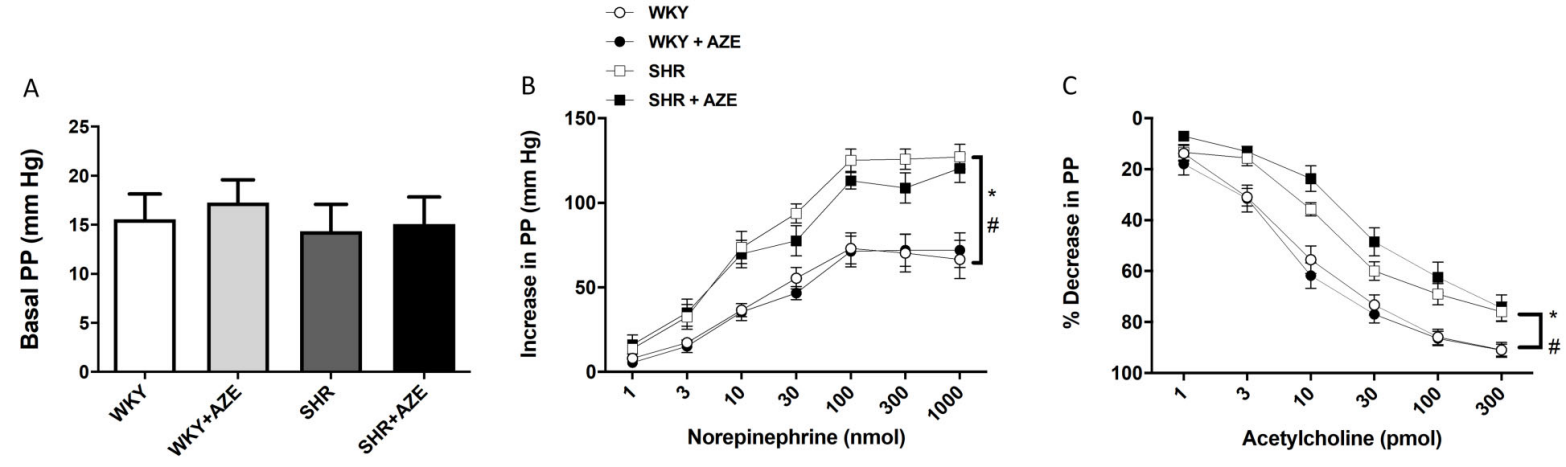
Vascular reactivity in mesenteric arterial bed. Basal perfusion pressure (PP) (**A**), vasoconstrictor response of norepinephrine (**B**), and vasodilator response of acetylcholine (**C**) in isolated mesenteric arterial bed. Data are reported as means±SE, n=7-10 per group. *P<0.05 *vs* WKY group; ^#^P<0.05 *vs* WKY+AZE (two-way ANOVA for basal PP and three-way ANOVA for acetylcholine and norepinephrine responses, followed by Sidak *post hoc* test). AZE: *Alpinia zerumbet* leaf extract; WKY: Wistar Kyoto rats; SHR: spontaneously hypertensive rats.

As shown in Figure 2A, the basal perfusion pressure did not differ among groups. However, the dose-dependent vasoconstrictor response induced by NE (1-1,000 nmol) in MAB was significantly higher in the SHR (P=0.00003) and SHR+AZE (P=0.0032) groups than in the respective controls (Figure 2B). ACh (1-300 pmol) induced a dose-dependent vasodilator response that was significantly lower in MAB from the SHR (P=0.024) and SHR+AZE (P=0.00007) groups than in the control groups (Figure 2C). AZE treatment did not improve vascular dysfunction in this hypertension model (Figure 2B and C).

### Effects of AZE on vascular and cardiac remodeling

**Figure 3:**
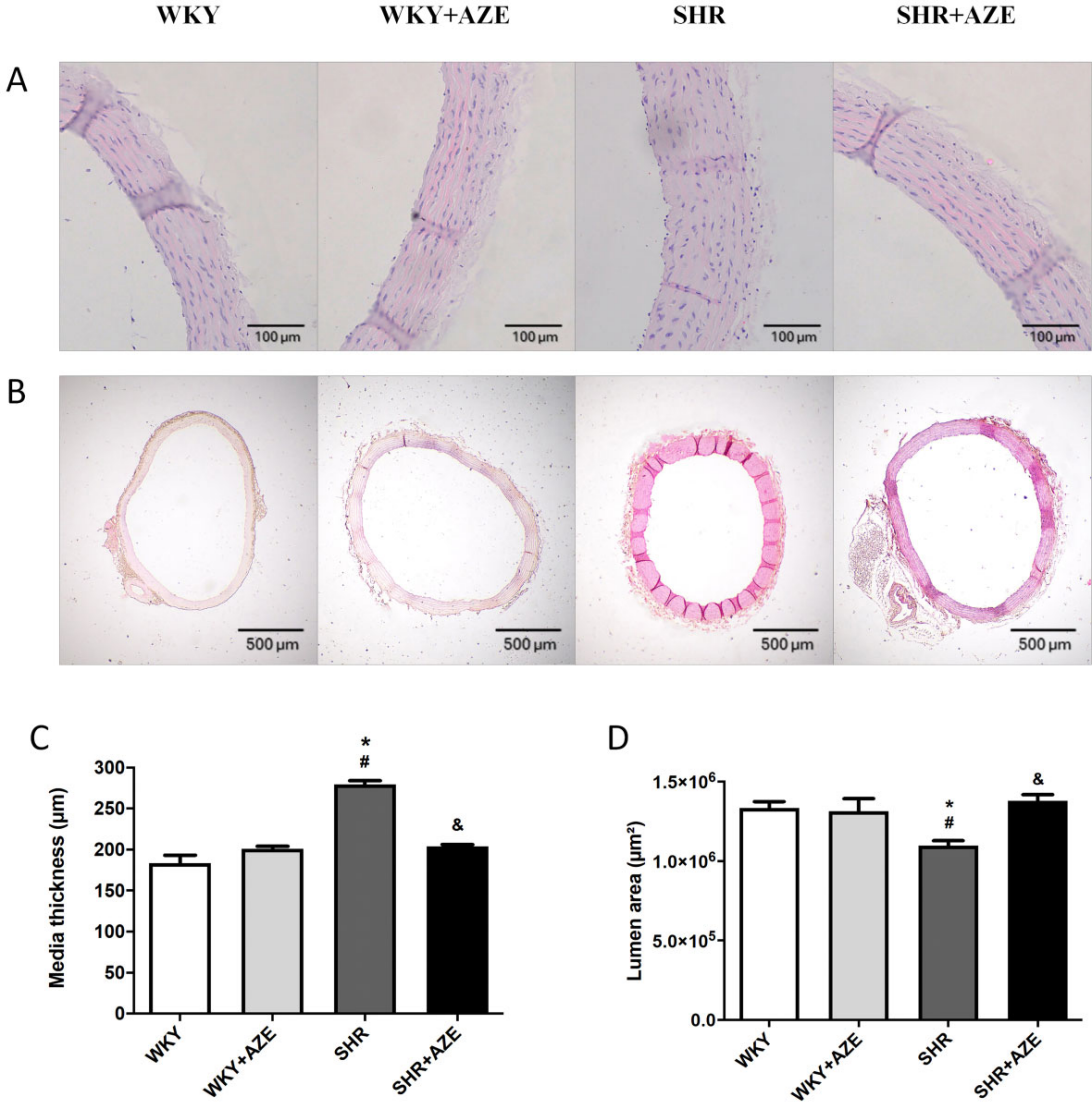
Thoracic aorta remodeling. Representative photomicrographs of thoracic aorta stained by hematoxylin and eosin (**A** and **B,** scale bars 100 and 500 μm), aorta media thickness (**C**), and lumen area (**D**). Magnification: **A** (×20); **B** (×4). Data are reported as means±SE, n=4 per group. *P<0.05 *vs* WKY group; ^#^P<0.05 *vs* WKY+AZE group; ^&^P<0.05 *vs* SHR group (two-way ANOVA followed by Sidak *post hoc* test). AZE: *Alpinia zerumbet* leaf extract; WKY: Wistar Kyoto rats; SHR: spontaneously hypertensive rats.

The SHR group presented a significant increase in the media thickness (P=0.000002; Figure 3A and C) and a reduction in the lumen area (P=0.043; Figure 3B and D) of the thoracic aorta compared to the control group, confirming the vascular remodeling in this model of hypertension. AZE treatment improved the histological changes in media thickness (P=0.000017) and lumen area (P=0.014) in the SHR+AZE compared to the SHR group (Figure 3).

**Figure 4:**
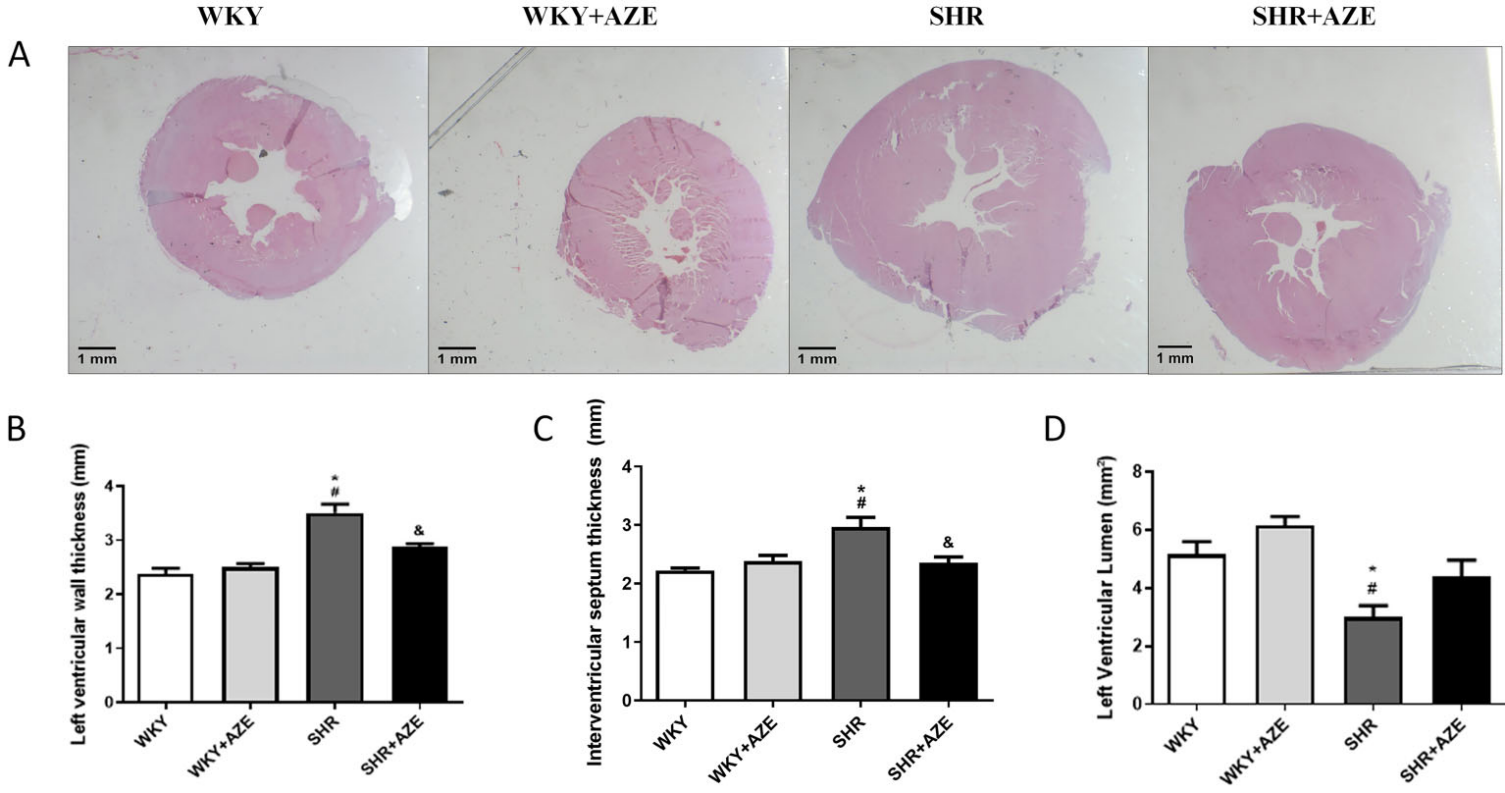
Left ventricle remodeling. Representative photomicrographs of the left ventricle stained by hematoxylin and eosin (**A**), left ventricle free wall thickness (**B**), interventricular septum thickness (**C**), and left ventricle lumen area (**D**). Magnification: ×0.5, scale bar 1 mm. Data are reported as means±SE, n=4-6 per group. *P<0.05 *vs* WKY group; ^#^P<0.05 *vs* WKY+AZE group; ^&^P<0.05 *vs* SHR group (two-way ANOVA followed by Sidak *post hoc* test). AZE: *Alpinia zerumbet* leaf extract; WKY: Wistar Kyoto rats; SHR: spontaneously hypertensive rats.

The SHR group presented cardiac hypertrophy (Figure 4A), characterized by an increase in the left ventricle (P=0.0001; Figure 4B) and interventricular septum (P=0.002; Figure 4C) wall thickness, as well as a decrease in the left ventricular lumen area (P=0.026; Figure 4D). The treatment with AZE restored the first two parameters (P=0.021 and P=0.011, respectively). However, it did not significantly modify the left ventricle lumen area (Figure 4).

**Figure 5:**
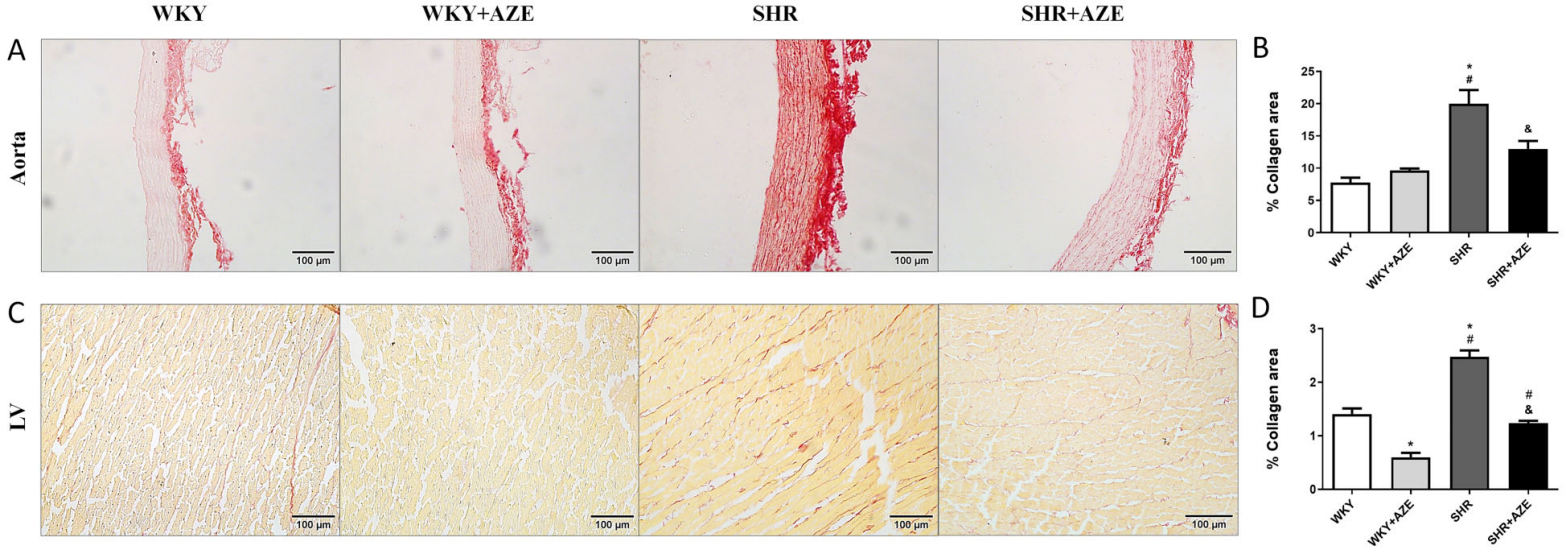
Fibrosis in the cardiovascular system. Representative photomicrographs of thoracic aorta stained by Picrosirius red (**A**) and collagen area (%) in thoracic aorta sections (**B**). Representative photomicrographs of the left ventricle stained by Picrosirius red (**C**) and collagen area (%) in left ventricle sections (**D**). Magnification: ×20, scale bar 100 μm. Data are reported as means±SE, n=4-5 per group. *P<0.05 *vs* WKY group; ^#^P<0.05 *vs* WKY+AZE group; ^&^P<0.05 *vs* SHR group (two-way ANOVA followed by Sidak *post hoc* test). AZE: *Alpinia zerumbet* leaf extract; WKY: Wistar Kyoto rats; SHR: spontaneously hypertensive rats; LV: left ventricle.

Pronounced collagen deposition in the aorta (tunica media) (P=0.00015; Figure 5A and B) and the left ventricle (interstitial fibrosis) (P=0.000002; Figure 5C and D) was observed in SHR compared to control groups. Collagen fibers were found intermingled among smooth muscle cells (aorta) and cardiomyocytes (LV). These detrimental changes were effectively restored by AZE administration (P=0.012 and P=0.0000002, respectively). AZE treatment also reduced collagen deposition in the left ventricle of WKY+AZE compared to WKY group (P=0.00006).

### Effects of AZE on Ang II levels and AT1R immunostaining

**Figure 6:**
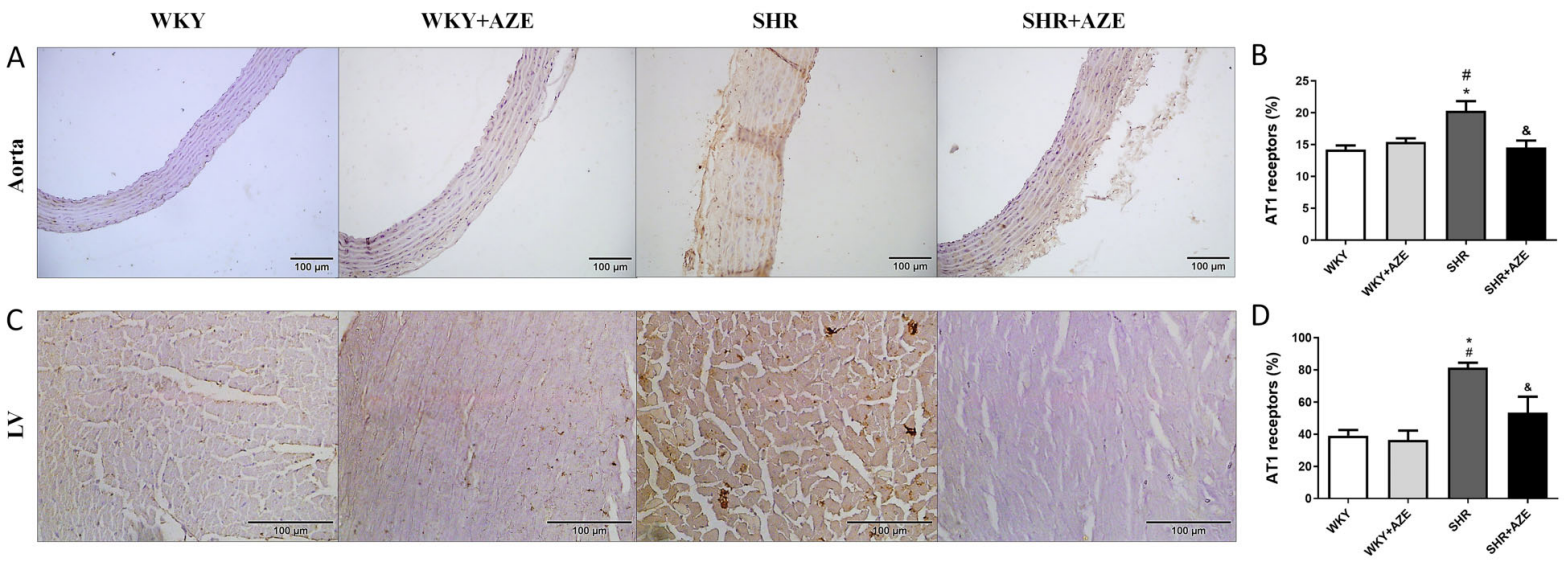
AT1 receptors in the cardiovascular system. AT1 receptor immunostaining (in brown) in the thoracic aorta (**A** and **B**) and left ventricle (**C** and **D**). Magnification: ×20, scale bar 100 μm. Data are reported as means±SM, n=4-5 per group. *P<0.05 *vs* WKY group; ^#^P<0.05 *vs* WKY+AZE group; ^&^P<0.05 *vs* SHR group (two-way ANOVA followed by Sidak *post hoc* test). AZE: *Alpinia zerumbet* leaf extract; WKY: Wistar Kyoto rats; SHR: spontaneously hypertensive rats; LV: left ventricle.

Although the levels of Ang II in plasma were similar among the experimental groups (Table 1), AT1R immunostaining was higher in the thoracic aorta (P=0.003; Figure 6A and B) and left ventricle (P=0.0009; Figure 6C and D) from SHR than those from control groups (P<0.05), and AZE treatment significantly reduced AT1 immunostaining in the thoracic aorta and left ventricle (P=0.009 and P=0.027, respectively) in the SHR+AZE compared to the SHR group (Figure 6).

**Table 1:** Effects of *Alpinia zerumbet* leaf extract (AZE) on body weight, Ang II levels, and oxidative status in SHR.

	WKY	WKY+AZE	SHR	SHR+AZE
Final body weight	342.2±5.3	351.5±8.8	332.0±10.2	332.0±9.1
Plasma				
Angiotensin II (pg/mL)	369.7±25.6	358.5±31.4	437.7±32.6	420.4±23.7
Malondialdehyde (nmol TBA/mg ptn)	0.35±0.04	0.26±0.03	0.28±0.02	0.20±0.02
Carbonyl (nmol/mg ptn)	419.3±64.8	378.5±67.0	991.2±73.2*^#^	636.6±86.8^&^
Superoxide dismutase (U/mg ptn)	40.0±2.9	43.1±7.4	23.1±4.6	23.3±4.8
Catalase (U/mg ptn)	0.52±0.08	0.39±0.05	0.09±0.02*^#^	0.42±0.08^&^
Glutathione peroxidase (U/mg ptn)	0.02±0.002	0.02±0.001	0.006±0.001*^#^	0.01±0.003^&^
Nitrite (µM)	123.8±17.2	110.5±9.9	68.6±6.5*^#^	93.4±9.0

Data are reported as means±SE (n=6-10 per group). *P≤0.05 compared with the WKY group (two-way ANOVA). ^#^P≤0.05 compared with the WKY+AZE group (two-way ANOVA). ^&^P≤0.05 compared with the SHR group (two-way ANOVA). WKY: Wistar Kyoto rats; SHR: spontaneously hypertensive rats; Ang II: angiotensin II.

### Effects of AZE on oxidative stress parameters and nitrite content

**Figure 7:**
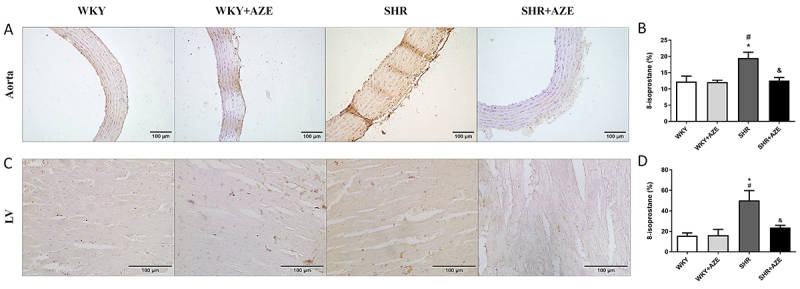
Lipid peroxidation in the cardiovascular system. 8-Isoprostane immunostaining (in brown) in the thoracic aorta (**A** and **B**) and left ventricle (**C** and **D**). Magnification: ×20, scale bar 100 μm. Data are reported as means±SE, n=4-5 per group. *P<0.05 *vs* WKY group; ^#^P<0.05 *vs* WKY+AZE group; ^&^P<0.05 *vs* SHR group (two-way ANOVA followed by Sidak *post hoc* test). AZE: *Alpinia zerumbet* leaf extract; WKY: Wistar Kyoto rats; SHR: spontaneously hypertensive rats; LV: left ventricle.

Oxidative damage was assessed by MDA and carbonyl levels in plasma samples (Table 1) as well as by 8-isoprostane immunostaining in the thoracic aorta (Figure 7A and B) and left ventricle (Figure 7C and D). Although MDA levels did not differ among groups, carbonyl levels were higher in the SHR group than in the controls (P=0.00004)). AZE treatment reduced (P=0.009) carbonyl levels in the SHR+AZE compared to the SHR group. 8-isoprostane immunostaining was higher in the thoracic aorta (P=0.008) and left ventricle (P=0.022) from SHR than in the control groups. AZE treatment improved this parameter in both tissues (P=0.011 and P=0.046, respectively).

**Figure 8:**
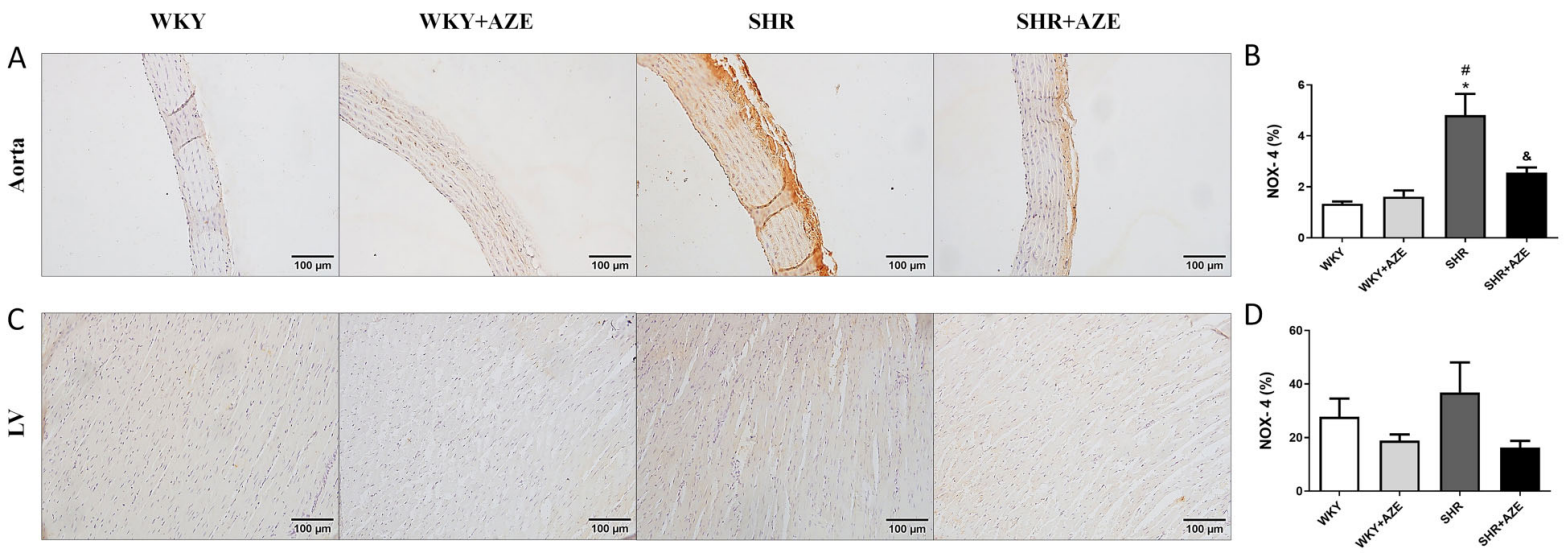
NOX-4 in the cardiovascular system. NOX-4 immunostaining (in brown) in the thoracic aorta (**A** and **B**) and left ventricle (**C** and **D**). Magnification: ×20, scale bar 100 μm. Data are reported as means±SE, n=5 per group. *P<0.05 *vs* WKY group; ^#^P<0.05 *vs* WKY+AZE group; ^&^P<0.05 *vs* SHR group (two-way ANOVA followed by Sidak *post hoc* test). AZE: *Alpinia zerumbet* leaf extract; WKY: Wistar Kyoto rats; SHR: spontaneously hypertensive rats; LV: left ventricle.

The immunostaining of NOX-4 (NADPH oxidase isoform - an important enzyme that generates reactive oxygen species) was increased in the thoracic aorta (Figure 8A and B) of animals from the SHR group compared to controls (P=0.0003). On the other hand, treatment of SHR animals with AZE reduced the expression of this enzyme compared to the non-treated SHR group (P=0.015). Regarding NOX-4 immunostaining in the left ventricle (Figure 8C and D), there was no significant difference between groups.

Superoxide dismutase, catalase, and glutathione peroxidase antioxidant activities were assayed in plasma samples (Table 1). Superoxide dismutase activity did not differ among groups. Catalase (P=0.002) and glutathione peroxidase (P=0.012) antioxidant activities were lower in the SHR group than in the controls. The treatment with AZE recovered the antioxidant activity of catalase (P=0.026) and glutathione peroxidase (P=0.018) in the SHR+AZE compared to the SHR group.

**Figure 9:**
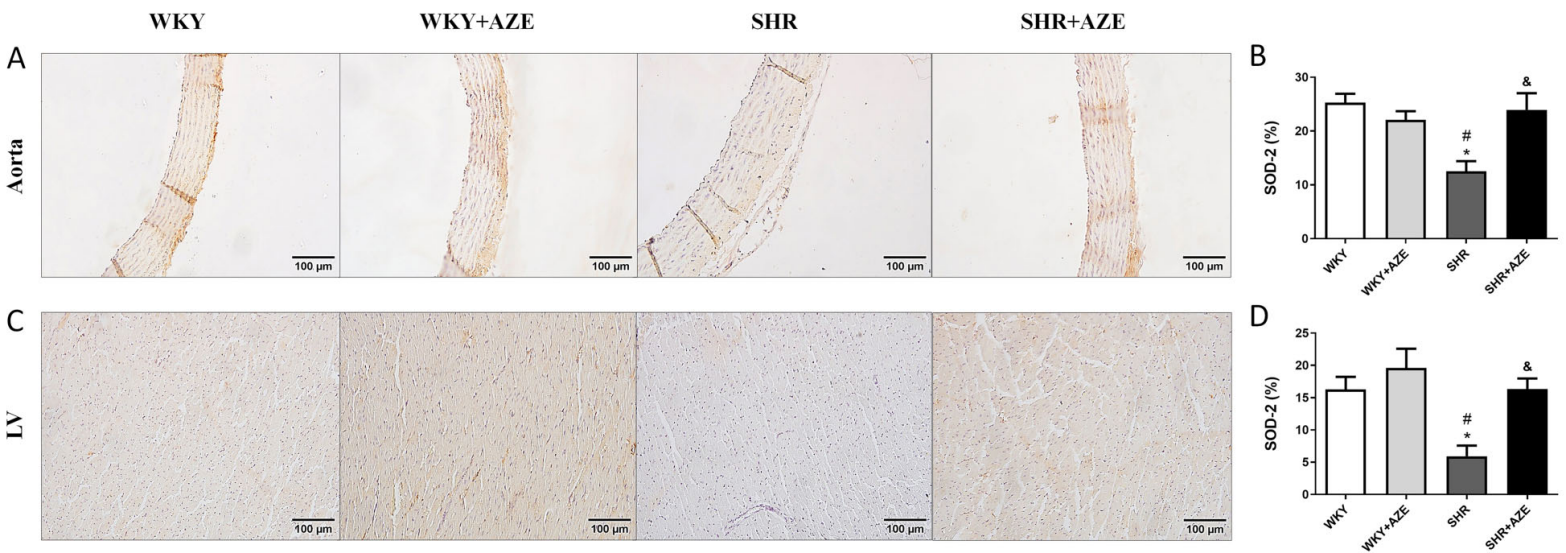
SOD-2 in the cardiovascular system. SOD-2 immunostaining (in brown) in the thoracic aorta (**A** and **B**) and left ventricle (**C** and **D**). Magnification: ×20, scale bar 100 μm. Data are reported as means±SE, n=5 per group. *P<0.05 *vs* WKY group; ^#^P<0.05 *vs* WKY+AZE group; ^&^P<0.05 *vs* SHR group (two-way ANOVA followed by Sidak *post hoc* test). AZE: *Alpinia zerumbet* leaf extract; WKY: Wistar Kyoto rats; SHR: spontaneously hypertensive rats; LV: left ventricle.

Furthermore, superoxide dismutase 2 immunostaining was reduced in the thoracic aorta (P=0.003; Figure 9A and B) and left ventricle (P=0.019; Figure 9C and D) of animals from the SHR group compared to controls. In contrast, AZE treatment significantly increased superoxide dismutase immunostaining in SHR+AZE compared to the SHR group in both tissues (P=0.01 and P=0.019, respectively).

Nitrite content, an indirect measure of NO, was lower in the plasma of animals from SHR groups than in the controls (P=0.007). AZE treatment did not modify this parameter (Table 1).

## Discussion

In this study, we evaluated the effects of the hydroalcoholic extract from *A*. *zerumbet* leaves on cardiovascular changes in SHR, an experimental model of essential hypertension.

Data from the present study demonstrated for the first time the ability of AZE to reverse hypertension in SHR. Our group previously demonstrated AZE's antihypertensive effect in DOCA-salt hypertensive rats (5). *A*. *zerumbet* is popularly used for its diuretic property. However, we did not observe increased diuresis in animals chronically treated with AZE in the present study. Therefore, the antihypertensive effect that we observed may not be related to a diuretic action of the extract. On the other hand, our group has previously reported AZE-induced vasodilation in MABs isolated from Wistar rats. This effect depends on the activation of the NO-GMPc pathway and might be modulated by bradykinin B2 receptors (5). Therefore, the AZE's vasodilator action may participate in its antihypertensive property. These findings support its indication as an antihypertensive medicinal plant.

The pathophysiology of hypertension is related to several factors, including genetics, activation of the sympathetic nervous system and RAS, inflammatory mediators, and endothelial dysfunction (26). The term endothelial dysfunction describes the altered metabolism of available NO or the imbalance of several endothelium-derived relaxing and constrictor factors (27). In the present study, SHRs demonstrated decreased acetylcholine-induced vasodilatation and increased norepinephrine-induced vasoconstriction in isolated MABs compared to normotensive rats. Additionally, plasma nitrite content, an indirect measure of NO, was lower in SHR than in controls, indicating decreased NO bioavailability. Taken together, these data confirmed the vascular dysfunction in this experimental model of essential hypertension (28). Although the treatment with AZE reduced blood pressure to normal levels, it was not effective in reversing endothelial dysfunction in this model.

The left ventricle is a primary target for hypertension end-organ damage, and LV hypertrophy is an independent risk factor for cardiovascular morbidity and mortality (29). Hypertensive heart disease involves the structural remodeling of the musculature and collagenous and non-collagenous matrix. In its initial stages, the hypertrophied ventricle can compensate in the face of an increased workload. However, in the later stages, the diastolic, and eventually, the systolic properties of the LV become impaired causing decompensation, and heart failure (30,31). Additionally, hypertension also leads to vascular remodeling, which involves changes to the vascular smooth muscle cell (VSMC) of both large and small arteries, and other cellular components of the vascular wall, including endothelial cells, elastin, and collagen content. During hypertension, conductive wall thickening and hypertrophy lead to increased vessel stiffness and decreased arterial compliance, which could negatively impact myocardial work capacity and coronary perfusion (32). Our present study demonstrated LV and thoracic aorta remodeling in SHR, corroborating data from the literature (33,34). Those cardiovascular changes were accompanied by increased collagen deposition. On the other hand, the treatment with AZE protected against cardiovascular remodeling in this model. It is important to mention that there was a reduction in collagen deposition in the LV of control animals treated with AZE, but this finding was not related to morphological changes in the tissue.

The precise mechanisms involved in AZE's beneficial effects on cardiovascular structural changes in SHR are unknown. We believe it is related to its antihypertensive and antioxidant actions, as demonstrated in the present study.

Different experimental models have demonstrated the relationship between oxidative stress and hypertension (35,36). Evidence points to lower activity of the antioxidant enzymes in the SHR (37). In this study, we observed increased oxidative damage (carbonyl group formation in plasma samples and 8-isoprostane immunostaining in LV and thoracic aorta) in SHRs. Increased oxidative damage in the SHR group was associated with impaired antioxidant defense in plasma, LV, and thoracic aorta and increased NOX-4 immunostaining in the thoracic aorta. Those data confirmed the oxidative stress in this model. In contrast, treatment with AZE reduced oxidative damage in SHRs, which was associated with improved plasma and tissue antioxidant defense and reduced NOX-4 in the thoracic aorta. Previous data have demonstrated the antioxidant effect of the essential oil extracted from the *A*. *zerumbet* leaves (38), but this is the first report on the improvement of oxidative stress induced by a hydroalcoholic extract obtained from *A*. *zerumbet* leaves. Thus, the AZE-induced antihypertensive effect and the impediment of cardiovascular remodeling may be related in part to its antioxidant action. A recent study from our group demonstrated that AZE is mainly composed of flavonoids, a group of natural substances with variable phenolic structures with significant antioxidant and chelating properties (13) that could contribute to the improvement of oxidative stress observed in the present study.

Considering the pro-oxidant action of Ang II and its essential role in cardiovascular remodeling (9-11), we investigated the circulating levels of this peptide and the tissue immunostaining of AT1R. Circulating levels of Ang II did not differ between the experimental groups, which is in agreement with a previous study (39). On the other hand, immunostaining of AT1R was increased in the LV and aorta of SHR animals, while AZE treatment reduced their tissue levels. Thus, the improvement in cardiovascular remodeling promoted by AZE may also be associated with the modulation of Ang II actions in the cardiovascular system.

In conclusion, we demonstrated that chronic treatment with AZE reversed hypertension and improved cardiovascular remodeling in the SHR model, which may be associated with its antioxidant actions and AT1R modulation. These findings suggested that AZE may be used for treating hypertension and related cardiovascular changes.
